# A Case of Epstein-Barr Virus Encephalitis and Orbital-Face Inflammation

**DOI:** 10.7759/cureus.56888

**Published:** 2024-03-25

**Authors:** Rui T Tang, Jose Gavito-Higuera, Claudia M Prospero Ponce

**Affiliations:** 1 Neurology and Ophthalmology, Texas Tech University Health Sciences Center El Paso Paul L. Foster School of Medicine, El Paso, USA; 2 Diagnostic and Interventional Imaging, McGovern Medical School UTHealth, Houston, USA; 3 Neurology and Ophthalmology, Texas Tech University Health Sciences Center El Paso, El Paso, USA

**Keywords:** epstein-barr virus, ocular and orbital infection, ophthalmoplegia, anisocoria, ptosis

## Abstract

Epstein-Barr virus (EBV) can cause follicular conjunctivitis, keratitis, oculoglandular syndrome, meningitis, and encephalitis. We report a 54-year-old Hispanic male who presented with right pupil-involved complete ophthalmoplegia, orbital and masticatory muscle inflammation, trigeminal enhancement, and new corneal infiltrate highly suggestive of EBV. Labwork was negative except for positive EBV polymerase chain reaction (PCR) in serum. Magnetic resonance imaging (MRI) of his brain and orbits with contrast showed enhancement of the right ganglion of the trigeminal nerve, oculomotor nerve, all extraocular muscles in the right orbit, and right masticatory and temporalis muscles and a right subacute lacunar infarct. The patient was diagnosed with encephalitis and orbital-face inflammation secondary to EBV infection. The patient improved with systemic steroids.

## Introduction

Epstein-Barr virus (EBV) is a ubiquitous double-stranded deoxyribonucleic acid (DNA) herpesvirus. After primary infection such as mononucleosis, it persists permanently in a latent state in B cells in the human body. Similar to other forms of viral encephalitis, EBV can also cause encephalitis with or without infectious mononucleosis, and it is characterized by fever, headache, confusion, seizure, and paresis. EBV encephalitis is more common in children [1,2]. EBV infection can manifest neurologically as encephalitis, aseptic meningitis, transverse myelitis, acute cerebellar ataxia, optic neuritis, Guillain-Barre syndrome, and acute demyelinating encephalomyelitis [2]. EBV can also cause ocular symptoms including follicular conjunctivitis, keratitis, and oculoglandular syndrome [3]. We present a case of adult corneal-orbital and extraorbital manifestation secondary to EBV infection.

## Case presentation

The patient is a 54-year-old Hispanic male with a past medical history of COVID-19 infection with one relapse, diabetes mellitus type 2, hypertension, hyperlipidemia, and sleep apnea and was admitted to the hospital due to painful ophthalmoplegia, facial pain, gingival numbness, and new onset of left foot drop, spastic leg, and need for a cane to walk. He had been admitted several times before due to right-sided headaches at outside hospitals and discharged with ineffective migraine treatment.

**Figure 1:**
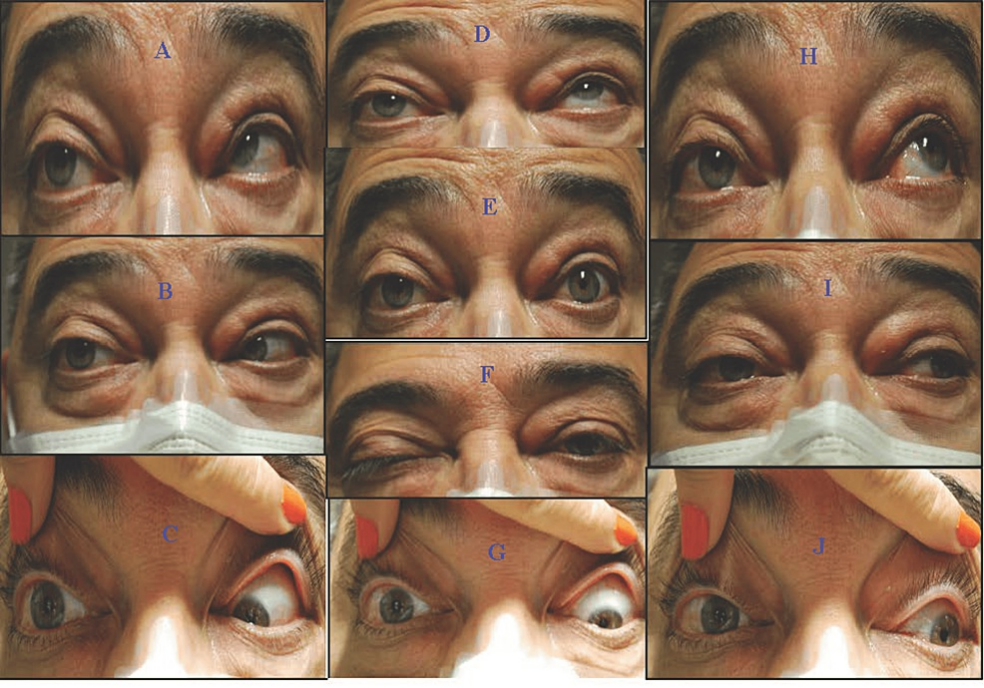
Extraocular motility assessment A: limited right and up gaze of the right eye (OD); B: limited abduction OD; C: limited right and down gaze OD; D: limited supraduction OD; E: anisocoria in the dark, the right pupil is larger; F: almost complete ptosis OD; G: limited infraduction OD; H: limited left and up gaze OD; I: limited adduction OD; J: limited left and down gaze OD. Additionally, on the exam (not shown), the right cornea had decreased sensation, and the eye showed resistance to retropulsion, which indicated inflammation.

**Figure 2:**
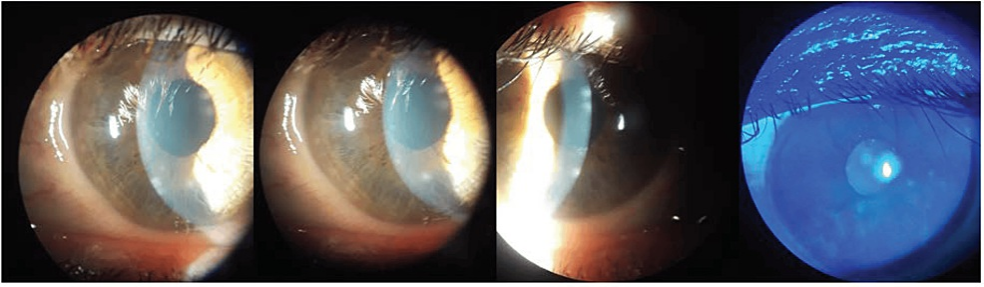
Slit lamp exam The slit lamp exam shows right corneal nummular white infiltrates in the anterior stroma with clear intervening stroma and quiet anterior chamber. Fluorescein shows no intake with cobalt blue light.

At the neuro-ophthalmology clinic visit, the patient reported incomplete resolution of intense burning pain on the right cheek, tingling and “ants crawling” sensation along the right inferior cheek and the superior temporal and parietal scalp, and diplopia. Visual acuity (VA), visual field (VF), and intraocular pressure (IOP) in both eyes (OU) were unremarkable. There was anisocoria, with the right eye dilated pupil poorly reactive to light or accommodation, but there is no relative afferent pupillary defect. Extraocular motor exam showed exotropia and ptosis of the right eye as well as ophthalmoplegia of the right eye (Figure 1). His right cornea also had decreased sensation during the exam. There was also resistance to retropulsion of the right eye, which indicated inflammation. The slit lamp picture showed right corneal nummular white infiltrates in the anterior stroma with clear intervening stroma and quiet anterior chamber. The infiltrates were negative to fluorescein staining (Figure 2). He denied dysphagia, facial weakness, visual obscuration, jaw claudication, or urinary or fecal incontinence but complained of worsening left lower extremity weakness.

**Figure 3:**
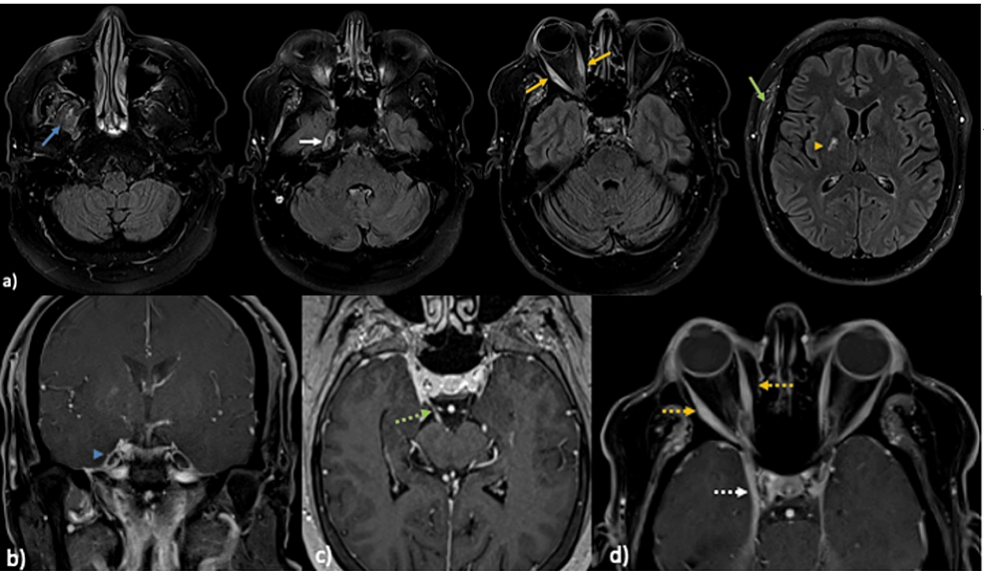
Brain and orbit MRI with contrast (a) Axial magnetic resonance imaging (MRI) T2 fluid-attenuated inversion recovery (FLAIR) images of the brain and orbits show asymmetric thickening and hyperintensity of the right-sided masticator muscles (blue arrow), the right ganglion of trigeminal nerve (white arrow), and all extraocular muscles in the right orbit (orange arrows) and right temporalis muscle (green arrow). There is an associated subacute lacunar infarct in the posterior limb or the right internal capsule (orange arrowhead). (b)-(d) Contrast MRI of the brain and orbit coronal T1 fat saturated with contrast (b), axial T1 fat saturated with contrast (c) and axial T1 fat saturated with contrast (d) showed abnormal asymmetric enhancement of the right ganglion of the trigeminal nerve (blue arrow head) and the right oculomotor nerve (green arrow), asymmetric thickening and enhancement of the right extraocular muscles (dotted orange arrows) and the right cavernous sinus (white dotted arrow).

The patient was admitted to the hospital. The temporal artery biopsy was normal. His motor strength was intact in all extremities; however, reflexes were diminished on the left lower extremity with positive Babinski’s sign. Axial magnetic resonance imaging (MRI) T2 fluid-attenuated inversion recovery (FLAIR) of the brain and orbit (Figure 3) showed asymmetric thickening and hyperintensity of the right-sided masticator muscles, the right ganglion of the trigeminal nerve, all the extraocular muscles in the right orbit, and the right temporalis muscle. There was an associated subacute lacunar infarct in the posterior limb or the right internal capsule. Magnetic resonance angiography (MRA) of the head was negative for cavernous sinus thrombosis. Spine MRI revealed moderate spinal canal and left neural foraminal stenosis at L4-L5 but no enhancement, which could explain his leg weakness. Full-body positron emission tomography (PET) scan and chest computed tomography (CT) were unremarkable. 

Laboratory workup (Table 1) was unremarkable except for the elevated erythrocyte sedimentation rate (ESR) (35) and high hemoglobin A1C (8.1%), positive herpes simplex virus (HSV) 1 IgG, positive herpes zoster virus (HZV) IgG. Quantiferon tuberculosis (TB), anti-cytomegalovirus, anti-Sjögren-syndrome-related antigen A/B, and anti-thyroid peroxidase antibody test (anti-TPO). Cerebrospinal fluid (CSF) analysis showed elevated white blood cells (40), protein (193), and glucose (93). Meningitis panel of CSF lyme, venereal disease research laboratory (VDRL), HSV polymerase chain reaction (PCR), HZV PCR, and EBV PCR were negative, but serum EBV PCR, EBV viral capsid antigen antibody, and EBV nuclear antigen came back positive four days later.

**Table 1:** Lab workup

Lab test	Pt value	Reference range
Erythrocyte sedimentation rate (ESR)	35 mm/h	0-15 mm/h (male), 0-20 mm/h (female)
Hemoglobin A1c	8.1%	<5.7%
Antinuclear antibody (ANA)	Negative	Negative
Bartonella antibodies	Negative	Negative
C-reactive protein (CRP)	<0.5 mg/dl	0-0.8 mg/dl
Florescent treponemal antibody absorption (FTA-ABS)	Negative	Negative
Rapid plasma reagin (RPR)	Negative	Negative
Human immunodeficiency virus (HIV)	Negative	Negative
Herpes simplex virus (HSV) 1 IgM	Negative	Negative
Herpes simplex virus (HSV) 1 IgG	Positive	Negative
Herpes zoster virus (HZV) IgG	Positive	Negative
Quantiferon tuberculosis (TB)	Negative	Negative
Anti-cytomegalovirus (anti-CMV)	Not detected	Not detected
Anti-Sjögren-syndrome-related antigen A antibodies (anti-SSA)	Negative	Negative
Anti-Sjögren-syndrome-related antigen B antibodies (anti-SSB)	Negative	Negative
Anti-thyroid peroxidase antibody (anti-TPO)	Negative	Negative
Cerebrospinal fluid/CSF analysis		
CSF white blood cells	40	<5
CSF protein	193	12-60 mg/dl
CSF glucose	93	40-70 mg/dl
Meningitis panel		
CSF lyme	Negative	Negative
CSF venereal disease research laboratory (VDRL)	Nonreactive	Nonreactive
CSF HSV	Not detected	Negative
CSF HZV	Not detected	Negative
CSF HZV polymerase chain reaction (PCR)	Not detected	Negative
CSF EBV PCR	Negative	Negative
EBV PCR serum	Positive	Negative
EBV viral capsid antigen antibody serum	Positive	Negative
EBV nuclear antigen serum	Positive	Negative

Because of the corneal findings and the diffuse orbital and extraorbital inflammation along with the CSF findings, a diagnosis of encephalitis and orbital-face inflammation secondary to EBV infection was made. The patient was initially treated with acyclovir, systemic steroids, and prednisolone 1% eye drops for the right eye and was then changed to only oral prednisone 80 mg, which significantly improved his symptoms and ophthalmoplegia without recurrence.

## Discussion

EBV infection is ubiquitous, as more than 90% of adults in the United States and worldwide have positive antibodies in their serum, yet they are asymptomatic [4]. Central nervous system (CNS) complications by EBV are relatively rare, especially among adults, compared to other viruses such as HSV, HIV, coxsackievirus, and enterovirus [1]. Specifically, studies have shown that EBV can cause both anterior segment eye diseases, including conjunctivitis, dacryoadenitis, episcleritis, keratitis, and iritis, and posterior segment eye diseases including retinitis, optic neuritis, and uveitis [3,5]. Furthermore, because EBV tends to infect mucosal surfaces and lymphoid tissues, past studies have reported ocular manifestations of EBV infection with a “salmon patch” appearance with multiple lymphoid follicles [3]. Interstitial nummular ring-shaped keratitis has been reported before in a patient with mononucleosis, although three forms of EBV keratitis have been reported in the literature [6]. 

Although the exact mechanism of EBV encephalitis is unclear, there are several proposed pathways [2]. Direct viral invasion can result in EBV encephalitis. The patient’s immune system can produce T-lymphocytes and antibodies that attack myelin-oligodendrocyte glycoprotein, which has a similar antigen to EBV. The main mechanism that results in EBV-related neurological symptoms is latent infection reactivation [2]. EBV encephalitis diagnosis requires a combination of molecular, serological, and imaging studies. To diagnose CNS complications by EBV, PCR is more sensitive in detecting active inflammation, and past comparison studies have shown that compared to positive IgM, which reaches its peak between the second and third weeks of acute infection [6], positive EBV PCR increases the chance of diagnosis by 16% [7]. The identification of serum antibodies directed against viral capsid antigen (VCA) can be used to diagnose acute EBV infection, whereas antibodies against both VCA and EBV nuclear antigen (EBNA) can be used to diagnose latent EBV infection [8]. Imaging study, especially MIR, is also useful. The characteristic MRI pattern for EBV encephalitis is increased T2 signal in the thalamus and basal ganglia, although diffuse signal intensity changes in either the gray or white matter can be seen [9]. Metagenomics is a new and plausible approach to diagnose EBV infections based on the development of next-generation sequencing technology [7].

Like other EBV symptoms, EBV ocular infections are self-limited in healthy individuals [6,7]. So far, no agents have been officially approved in the United States or Europe to treat acute or chronic EBV infection [8]. There is no standard treatment for EBV encephalitis, although past studies have shown that patients usually recovered after receiving acyclovir and high-dose steroid therapy, except in immunocompromised patients [6]. If there is active inflammation of the eye, topical prednisolone or phosphate 1% one drop four times a day has also been recommended [5].

## Conclusions

In conclusion, our case report highlights the clinical signs and symptoms of EBV infections with concomitant involvement of the corneal, orbital, and central nervous system. Our case also highlighted our approach to reaching the diagnosis, including extensive laboratory and imaging workup (e.g., MRI, PET scan, and CT), and ruled out other differential diagnoses including herpes, syphilis, lyme disease, TB, meningitis, sarcoid, and giant cell arteritis. With a positive EBV PCR in the serum and pleocytosis and high protein in the CSF, we finally diagnosed the patient with encephalitis and orbital-face inflammation secondary to EBV infection. Because EBV ophthalmic infection is uncommon, our case underscores the importance of having a wide differential diagnoses in the beginning. Our patient was treated and improved with oral acyclovir and systemic steroids. Future studies may be warranted to examine why certain adults are more vulnerable to EBV-related ophthalmic and CNS infections, despite that EBV infection is ubiquitous. Overall, this case contributes to the existing literature on EBV ophthalmic and CNS infections in adults and its workup process. 
